# High-Throughput Sequencing of Three *Lemnoideae* (Duckweeds) Chloroplast Genomes from Total DNA

**DOI:** 10.1371/journal.pone.0024670

**Published:** 2011-09-09

**Authors:** Wenqin Wang, Joachim Messing

**Affiliations:** Waksman Institute of Microbiology, Rutgers, The State University of New Jersey, Piscataway, New Jersey, United States of America; J. Craig Venter Institute, United States of America

## Abstract

**Background:**

Chloroplast genomes provide a wealth of information for evolutionary and population genetic studies. Chloroplasts play a particularly important role in the adaption for aquatic plants because they float on water and their major surface is exposed continuously to sunlight. The subfamily of *Lemnoideae* represents such a collection of aquatic species that because of photosynthesis represents one of the fastest growing plant species on earth.

**Methods:**

We sequenced the chloroplast genomes from three different genera of *Lemnoideae*, *Spirodela polyrhiza*, *Wolffiella lingulata* and *Wolffia australiana* by high-throughput DNA sequencing of genomic DNA using the SOLiD platform. Unfractionated total DNA contains high copies of plastid DNA so that sequences from the nucleus and mitochondria can easily be filtered computationally. Remaining sequence reads were assembled into contiguous sequences (contigs) using SOLiD software tools. Contigs were mapped to a reference genome of *Lemna minor* and gaps, selected by PCR, were sequenced on the ABI3730xl platform.

**Conclusions:**

This combinatorial approach yielded whole genomic contiguous sequences in a cost-effective manner. Over 1,000-time coverage of chloroplast from total DNA were reached by the SOLiD platform in a single spot on a quadrant slide without purification. Comparative analysis indicated that the chloroplast genome was conserved in gene number and organization with respect to the reference genome of *L. minor*. However, higher nucleotide substitution, abundant deletions and insertions occurred in non-coding regions of these genomes, indicating a greater genomic dynamics than expected from the comparison of other related species in the *Pooideae*. Noticeably, there was no transition bias over transversion in *Lemnoideae*. The data should have immediate applications in evolutionary biology and plant taxonomy with increased resolution and statistical power.

## Introduction

Plants are defined by primary plastids, encompassing algae, Streptophytes, and land plants [1]. Each plant cell has three genomes, separated in three subcellular compartments, the nucleus, the chloroplasts, and the mitochondria. Chloroplasts are key organelles of green plants for photosynthesis. They are also responsible for storage of starch, and synthesis of chlorophyll, nucleic acids, and 50% of soluble protein in leaves. Chloroplasts are highly conserved in terms of their structure, genome size (from 120 to 217 Kb) and its gene content (∼130 genes) [2]. Typically chloroplast genomes in plants contain two identical inverted repeats (IRa and IRb), separated by unique sequences, the large single copy (LSC) and the small single copy (SSC) regions [3]. Chloroplasts contain multiple copies of a circular, double-stranded DNA molecule. For instance, leaf cells of tobacco and pea typically have ∼100 chloroplasts and up to 10,000 DNA copies [4]. Total genomic DNA could have as much as 5,000 times the copies of chloroplast DNA relative to nuclear gene copies as tested in monocots and dicots [5]. In addition to its important biological roles, chloroplast genome sequences are widely used in evolutionary studies, comparative genomics [6], and biotechnology [7].

*Lemnoideae* (duckweeds) are a subfamily of the *Araceae* of aquatic flowering monocot plants [8]. However, their minute size and simple morphologically characteristics made them extremely difficult for systematic analysis and species identification. Integration of morphological, flavonoid, allozyme, and DNA markers have yielded a single and well-resolved maximum parsimonious tree, but the resolution for closely related species is problematic with very low value of bootstrap support [9]. The same is true for DNA barcoding of the *Lemnoideae* subfamily. Actually, the *atpF*-*atpH* marker appeared to be the most powerful barcode to distinguish individual species of *Lemnoideae* with 14 out of 19 species, still short of complete coverage [10]. Indeed, a prevalent feature of chloroplast genomes is their high degree of sequence conservation. Choices of greater numbers of divergent sequences should increase resolution both for the exploration of plant relationships and DNA plant barcoding. Because the chloroplast genome in contrast to the nuclear genome is haploid and is uniparentally inherited, acting as a single locus, it has the potential to become the elusive universal single-locus for plant species identification and systematic analysis.

Duckweeds also have great potential industrial applications. Their biomass doubles every 1 or 2 days. They contain a starch content of 45.8% (dry weight) growing in wastewater [11]. They can keep accumulating starch as high as 65% when switching from frond to the turion phase [12]. Therefore, duckweeds have been proposed as an alternative starch source for fuel production. Taking into account the recent improvements in transplastomic techniques, which provides an environmentally benign method of plant genetic engineering and accumulates extraordinarily high levels of foreign proteins [7], duckweed chloroplast transformation would greatly accelerate the exploration of its biofuel potential.

Traditionally, chloroplast genomes have been sequenced by primer walking based on closely related known genomes [13] or by shotgun sequencing [6]. However, with the advent of next generation sequencing platforms a new cost-effective option to capture multiple genomes on a larger scale has arisen [14]. Still, the separation of plastid DNA from nuclei and mitochondria can be tedious and would require the use of multiple long PCR reactions to obtain overlapping fragments (5 to 10 Kb) of the entire chloroplast genome, which could produce long gaps if some PCR reactions would fail [14], [15]. Another way is to use a modified chloroplast isolation protocol and further amply them by multiple-primed rolling circle methods [16]. Either way, it would need substantial efforts to obtain enriched chloroplast DNA that could contain significant amounts of contaminating non-target DNA.

A recent study reported that chloroplast genome sequences were recovered from total DNA including nuclei, chloroplasts, and mitochondria by using an Illumina-based sequencing platform. Still, many gaps could not be bridged because of highly divergent regions [17]. However, here we could demonstrate that it is possible to assemble complete chloroplast genome sequences from total leaf DNA with the SOLiD sequencing platform at a high level of accuracy, following the same principles that have been applied to the first genome assembled entirely by shotgun DNA sequencing [18]. To obtain regions from the chloroplast genome that diverged from a reference genome, *de novo* assembly was employed using paired reads based on the concept of universal synthetic primers [19]. Before assembly, SOLiD reads from mitochondrial and nuclear DNA, were filtered electronically. Furthermore, we could use the chloroplast genome of the closely related species *L. minor* as a reference that has been sequenced with traditional overlapping long reads [13]. Genome assembly, the comparative and phylogenetic analyses of these genomes are presented here.

## Methods

### DNA isolation and SOLiD DNA sequencing

Duckweeds sequenced in this study (Table 1) were grown from a cluster of 3–5 fronds produced by a single mother frond. Total DNA was extracted from whole plant tissue by the CTAB method [20]. Sequencing runs were done on a SOLiD™ 3 Analyzer (Applied Biosystems, Foster City, CA) at the Waksman Genomics Core Facility of Rutgers University. Mate-paired libraries with approximately 1.5 Kb inserts were constructed from 20 µg of genomic DNA following the manufacturer's instructions (SOLiD sample preparation protocol for Mate-Paired library sequencing), and deposited in one spot of a quadrant slide. Fifty nucleotide-long reads were obtained from each of the F3 and R3 tags, with more than 100 million reads obtained for each of the genomes.

**Table 1:** Species used for comparative genomic analysis.

Species	Source	Nuclear Genome Size^b^ (Mbp)	Chloroplast Genome Size (bp)	Inverted Repeats Size (bp)	Genbank Number
*Spirodela polyrhiza* 7498	North Carolina, Durham Co., Durham, 'USA	160	168788	31755	JN160603
*Lemna minor* (reference) a	Russia	356-604	165955	31223	DQ400350
*Wolffiella lingulata* 7289	Amazonas, Manaus, Rio Negro, 'Brazil	655	169337	31683	JN160604
*Wolffia australiana* 7733	Mount Lofty Range, Torrens Gorge, 'South Australia	357	168704	31930	JN160605

a Reference chloroplast genome [13];

b Nuclear genome sizes [29].

### Sequence data analysis pipeline

**Figure 1:**
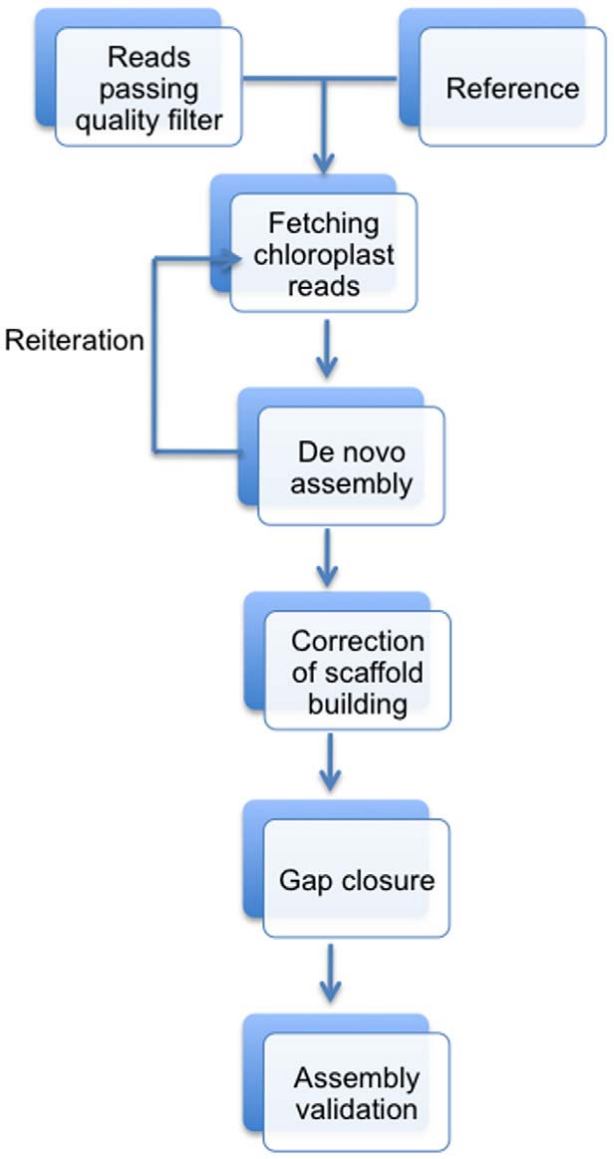
Pipeline of chloroplast genome assembly. Details are described under Methods.

To assemble the chloroplast genomes using SOLiD reads and close the remaining gaps with long reads from capillary electrophoresis (CE) sequencers, we used the following steps (Fig. 1). Because all chloroplast genomes contain two identical inverted repeats (IRs), we first assembled genomes without IRb's and with LSC, SSC, IRa, but added them later on for the full-length molecules.

1) Data filtering: SOLiD mate-paired short reads were preprocessed by Mean Filter of a Perl script [21]; i.e., reads were truncated to 40 bp and average quality of reads were set to exceed the threshold QV score of 20. Because coverage is very high, only successful mate-pair reads went into the next step. 2) Selection of chloroplast-related reads: The filtered mate-pair colorspace reads from each of the three samples were aligned to the chloroplast genome of *L. minor* [13] (GenBank accession number: DQ400350) using the BWA short-read alignment component with default parameters [22]. At least one end of the paired-end reads was anchored to the chloroplast genome of *L. minor* before interrogating the second end to map to a linked sequence or to a gap. 3) 1^st^ run of genome assembly: *De novo* assembly was performed with identified chloroplast-related reads using the SOLiD™ System *de novo* Accessory Tools 2.0 (http://solidsoftwaretools.com/gf/project/denovo/) in conjunction with the Velvet assembly engine [23]. These tools are designed to simplify and optimize parameters for ease of use and best performance. They sample an optimal sub-set of reads and automatically estimate optimal parameters for each step. Velvet parameters generated from the tools were deposited in Table 2 with hash length 19 and coverage cut-off 11. The assembly assistant module in the tool kit took the input from Velvet and produced scaffolds with 120 mate-pair confirmations to make confident scaffolding at the conclusion of this pipeline. 4) 2^nd^ run of genome assembly: After the first run, all scaffolds were concatenated into pseudomolecules. In order to maximize chloroplast-related reads, the artificial molecule functioned as a new reference and step 2 and 3 were then reiterated. 5) Correction of scaffold building: The biggest scaffolds of each genome were aligned with the most closely related reference genome of *L. minor* using BLAST2 (http://blast.ncbi.nlm.nih.gov/). Indeed in a few instances, non-contiguous genomic regions were found in juxtaposed positions at gap positions. At these gaps scaffolds were broken and contigs reordered in collinearity with the reference genome. Smaller contigs were manually ordered based on the reference genome. All scaffolds were then concatenated into a single full-length molecule, where each gap in the sequence was marked with one N. 6) Gap closure: Gaps were small enough so that flanking primer pairs could be chosen (Table S1) to isolate missing sequences by PCR and apply CE sequencing methods (ABI 3730XL) for closure. PCR amplification and conditions have been described recently [10]; 7) Assembly validation: Because PCR amplification of gaps required correct ordering of contigs into scaffolds, the long CE reads provided validation of overlapping sequences and the correct ordering of short read assemblies. Accumulative overlaps and discrepancies between alignments of sequences from both methods were summarized using DNASTAR software (http://www.dnastar.com/), which would reveal sequencing errors of the SOLiD platform. Because of mate-pair data junctions between IRb and LSU or SSU could be confirmed with CE sequencing of PCR products. 8) GenBank deposition: The fully sequenced genomes of the three species were annotated by DOGMA [24], checked manually, and have been deposited into GenBank as a whole genome shotgun project (Table 1).

**Table 2:** *De novo* assembly statistics for the three sequenced species.

Species	Read processing	Sc	N50 Sc (bp)	Co	N50 Co (bp)	Co length (bp)	Hash length	Expected coverage	Coverage cut-off^a^	min_pair_count^b^	Total reads (X10∧6)	Aligned reads (%)	Average genome coverage	Average nuclear coverage
*Spirodela polyrhiza* 7498	with selection	3	92558	60	4246	136597	19	150	11	120	153	12.9	5474	42
	without selection	6	36267	73	4062	132134	19	150	30	120				
*Wolffiella lingulata* 7289	with selection	1	136457	53	4708	139523	19	150	11	120	155	2.5	1070	12
	without selection	8	25221	82	2333	133615	19	150	6	120				
*Wolffia australiana* 7733	with selection	2	134892	39	8677	137183	19	150	11	120	111	6.2	1912	15
	without selection	3	98687	60	3743	132446	19	150	11	120				

a Coverage cut-off: minimum coverage required to form a contig.

b min_pair_count: number of mate-pair confirmations required for confident scaffolding. Sc = scaffold; Co = contig.

To assess the contribution of the filtering step with the reference genome to the performance of Velvet as an assembly tool, we also performed an assembly with total DNA reads including the nuclear and mitochondria DNA. Under these conditions, we could not use the default set-up parameters for the pipeline, which requires uniform coverage by a single genome. Otherwise, the precomputed parameters would extract sub-set reads that represent a mixture of three genomes with different coverage. To avoid this, we determined the optimized parameters after omitting data filtering as step 1 by empirically testing parameters for step 2, 3, and 4 and then manually accessing the SOLiD™ System *de novo* Accessory Tools 2.0 as shown in Table 2. All other assembly steps were the same as described with selected reads.

### Whole genome alignments, comparison, and phylogenetic analysis

*Lemnoideae* chloroplasts, *S. polyrhiza* 7498 (S.pol), *L. minor* (L.min), *W. lingulata* 7289 (W.lin), *W. australiana* 7733 (W.aus) were aligned by a program of global multiple alignments of finished sequences (Multi-LAGAN) [25] and annotation for the reference genome of *L. minor* [13] was used to construct sequence conservation plots in the program mVISTA [26].

The 81 protein coding nucleotide sequences from duckweeds were retrieved after annotation by DOGMA, concatenated as one full-length molecule and pair-wisely aligned with each other by Multi-LAGAN. MEGA 5 was used to detect transitions, transversions, and INDELs (insertion/deletion) for all genomes except the IRb regions and protein coding sequences. A similar analysis of 71 common genes was done for chloroplast genomes of species in the subfamily of the *Pooideae*, i.e., wheat (AB042240), barley (EF115541) and *Brachypodium* (EU325680). They were chosen because wheat and barley belong to the same tribe of *Triticeae*, whereas *Brachypodium* belongs to the different tribe of *Brachypodieae* within the same subfamily. This is taxonomically equivalent to the division within the subfamily of the *Lemnoideae.* The *Spirodela* and *Lemna* species belong to the same tribe, but *Wolffiella* and *Wolffia* to a different one [8], [9].

To examine whether the genome-wide phylogenetic analyses were consistent with those of morphological, flavonoid, and allozyme markers, as well as selected DNA sequences [9], we employed Maximum Parsimony to reconstruct the *Lemnoideae* phylogeny with whole chloroplast sequences by using MEGA 5 [27]. *Phoenix dactylifera* is in the same class of *Liliopsida* as *Lemnoideae* and functions as an outgroup here [28]. However, one of the two inverted repeat regions (IRb) was excluded from phylogenetic analyses.

## Results

### *De novo* assembly of short sequence reads yields high quality contigs

The chloroplast genomes of *S. polyrhiza*, *W. lingulata* and *W. australiana* in this study were selected on the basis of phylogenetic diversity of the subfamily *Lemnoideae* and their extensive variation of nuclear genome sizes (Table 1) [29]. The three genomes were sequenced using mate-paired libraries with the SOLiDTM 3 System. The previously sequenced *L. minor* chloroplast genome was used as computational filter to separate chloroplast reads from nuclear and mitochondria reads. Considering the identical feature of the two inverted repeats, we first assembled 136 Kb of the chloroplast genome from the LSC, IRa, SSC regions. All three genomes were each processed into one single large scaffold of 92 Kb (S.pol), 136Kb (W.lin), and 134 Kb (W.aus), respectively. Assembly of SOLiD reads resulted between 39 to 60 contigs and 1 to 3 scaffolds per genome (Table 2). With the second largest scaffold of 40 Kb for S.pol, the length of all the added contigs already reached a size expected for a chloroplast genome excluding the IRb region. However, alignment of these assemblies with the reference genome suggested between one to three misassembled scaffolds that needed to be corrected. Most contigs were interrupted by mononucleotide repeats and low complexity sequences.

Clearly, read length is a critical factor for assembly programs, but how critical is the filtering step for separating the mixture of nuclear, mitochondria, and chloroplast genomic sequences for the assembly tool used here. We therefore modified the parameters and the steps in the data processing protocol empirically to produce sequence assemblies without prior selection of chloroplast sequences. *De novo* assembly from total reads generated 60–82 contigs with 2333–4062 bp of N50 contig length, whereas assembly from selected chloroplast reads gave us a significant improvement with 18% to 35% lower contig numbers and longer N50 values of contig lengths (Table 2). If the computational read selection were omitted, 13–29 additional PCR reactions would have been required to close the gaps from total reads assembly and validate order of contigs and scaffolds as described below.

**Figure 2:**
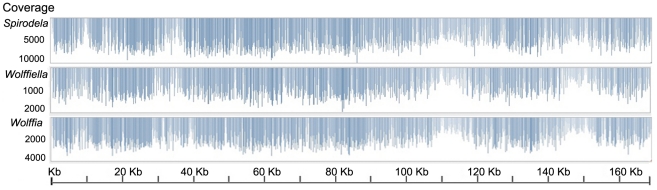
Coverage of *Lemnoideae* chloroplast genome by SOLiD system reads. Depth of coverage was plotted along the genome coordinates. Blue peaks show the coverage.

Using the ends of contigs separated by Ns, primers were designed for PCR amplification. Because of the alignment with the reference genome, the correct ordering of contigs could be confirmed by the fact that PCR amplification occurred. Furthermore, when PCR products were sequenced by the CE ABI 3730XL platform, overlapping sequences could be used to close gaps and validate the order of contigs. Accumulative overlaps for the three genomes totaled 48 Kb. When short read assemblies were compared with CE long read sequences, the cumulative differences amounted to just 0.041%, reflecting a high consensus between the two sequencing methods. We also could test the short read assembler by mapping *de novo* assemblies back to the complete genome. Although only 2.5∼12.9% of the reads were successfully aligned, keeping in mind the DNA mixture from plant tissue, this was sufficient to give a mean coverage between 1,070 to 5,474 times (Table 2 and Fig. 2). The IRa and IRb regions had lower coverage due to random placement of repetitive read pairs when mapping. For nuclear genome sequences, we found 12 to 42-fold coverage by ignoring mitochondrial DNA reads (Table 2). Based on these assessments, there were approximately 100 chloroplast genome copies for every nuclear genome copy.

### Sequence comparison and phylogeny among *Lemnoideae* chloroplast genomes

**Figure 3:**
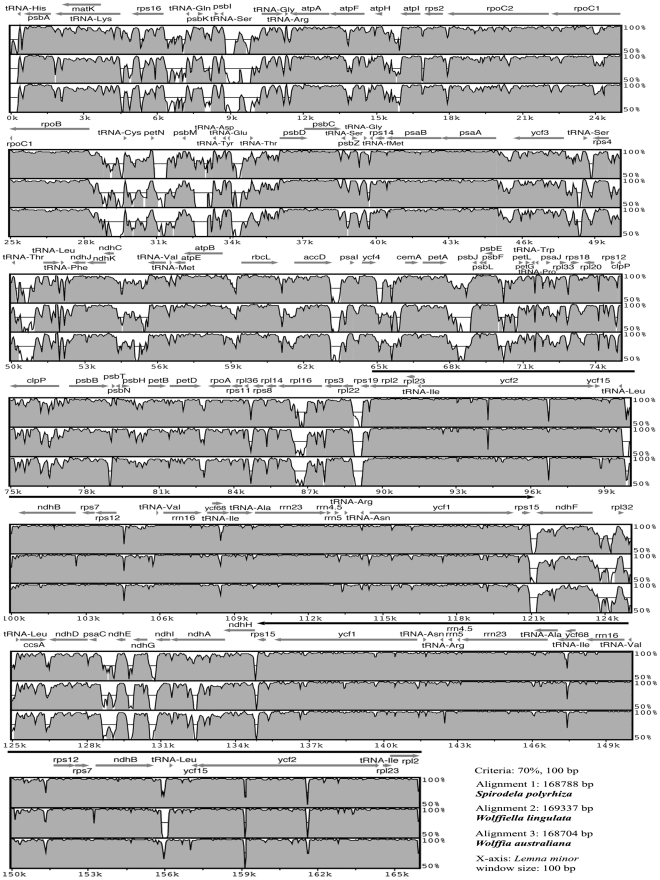
Alignment of *Lemnoideae* chloroplast genomes. The sequence of *L. minor* chloroplast genome was compared to those of *S. polyrhiza* (top), *W. lingulata* (middle), *W. australiana* (bottom). Sequences were aligned in mVISTA and the annotation shown above the alignment corresponds to the *L. minor* genome. Grey arrows above the alignment indicate genes and their orientation. Thick black lines show the position of the IRs. The grey peaks determine the percent identity between two sequences of *L. minor* as the reference and our sequenced genomes.

The chloroplast genomes of duckweeds appeared to be within a short range of 165,955 bp to 169,353 bp in length (Table 1). All of them include a pair of inverted repeats of around 31 Kb separated by SSC and LSC. Large single copy (LSC) and Small Single Copy (SSC) regions were close to 90 Kb and 10 Kb long, respectively. *S. polyrhiza*, *W. lingulata* and *W. australiana* contain the same gene number and order as the reference genome *L. minor* (Fig. 3).

The conservation of the overall structure of the chloroplast genomes allowed us to align the sequences of four duckweed species at the genome-wide level. Comparison of the sequences revealed multiple hotspots of high sequence length polymorphism (Fig. 3). The IRs showed lower sequence divergence than the single-copy regions. The majority of highly divergent regions were in non-coding regions as illustrated in an mVISTA alignment plot. The region between *rpoB* and *psbD* from position 28 Kb to 36 Kb is one of the most polymorphic regions. For example, *W. australiana* has a 425-bp deletion in the 29 Kb *rpoB*-tRNA-Cys region. *S. polyrhiza* has a 505-bp deletion compared with 100-bp deletions in *W. lingulata*, whereas a 353-bp insertion occurred at 31 Kb of the intergenic *petN*-*psbM* region of *W. australiana*. Both *W. lingulata* and *W. australiana* have a 460-bp deletion in the 32 Kb *psbM*-tRNA-Asp region. Moreover, some INDELs existed in introns, such as a 123-bp insertion in *atpF* of *Spirodela* at 13 Kb, and 114-bp deletion in *ndhA* for *W. lingulata* and 105-bp for *W. australiana* at the 132 Kb region (Fig. 3).

**Figure 4:**
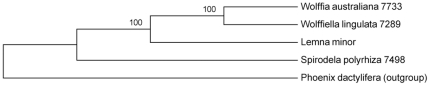
Complete chloroplast genome phylogeny of *Lemnoideae*. The phylogram was drawn by Maximum Parsimony with 1000 replicates of bootstrap test. The tree was rooted by *Phoenix dactylifera* as an outgroup. Support from bootstrap value was shown at the nodes. The GenBank accessions used for the analyses are JN160603 (*S. polyrhiza*), DQ400350 (*L. minor*), JN160604 (*W. lingulata*), JN160605 (*W. australiana*) and GU811709 (*P. dactylifera*). The whole genome sequences were aligned by Multi-LAGAN and MEGA 5 was used to draw the tree.

Maximum parsimony produced a single fully resolved tree with strong node support (Fig. 4). Our phylogenetic results showed *Wolffiella* and *Wolffia* were more closely related than the others. Furthermore, our analysis strongly supported that *Spirodela* was at the basal position of the taxon, followed by *Lemna* and *Wolffiella*, whereas *Wolffia* was the most derived (Fig. 4).

### Evolution of *Lemnoideae* and *Pooideae*, with chloroplast genomes in different orders

To further evaluate the pace of evolutionarily divergence, we compared chloroplast genomes from different monocot orders by quantifying nucleotide substitution rates and INDELs ratios. The subfamily of *Pooideae* within the *Poaceae* belongs to the order of the *Poales*, whereas the *Lemnoideae* belong to the order of the *Alismatales*. When such a comparison is made, duckweeds have a higher rate of substitution than species of the *Pooideae* at the whole genome level and in protein-coding regions. Moreover, INDELs were very prominent in duckweed genomes with ratios of 0.061 to 0.095, whereas they were much higher than the values between 0.006 and 0.012 in conservative coding regions. When we compared duckweeds with species of the *Pooideae*, duckweeds had twice as many INDELs in their chloroplast genomes than the *Pooideae’s* species based on the same level of intra-tribe or inter-tribe comparisons (Table 3). Based on INDELs length in genome and coding regions (Table 3), we could conclude that most INDELs were located in non-coding regions. Interestingly, we found that transversions were higher than transitions in the subfamily of *Lemnoideae* with R-values from 0.6 to 0.7 of the total genome. The same result was discovered in protein coding regions except between *S. polyrhiza* and *L. minor* (R = 1.1). However, these values were completely the opposite in the species of the subfamily of *Pooideae* with R-values from 1.2 to 1.7, where transitions were more numerous than transversions (Table 3).

**Table 3:** Pairwise sequence divergence of the whole genome and protein coding regions in the subfamily *Lemnoideae* compared with those of the subfamily *Pooideae* (wheat, barley and *Brachypodium*).

Comparative Type	Alignment Region	Pair Alignment	Alignment Length	Substitution Rate^a^	R = si^b^/sv^c^	INDELs Length	INDELs Ratio^d^
intra-tribe	whole genome	S.pol+L.min	141014	0.05	0.7	10262	0.073
intra-tribe	whole genome	W.lin+W.aus	141506	0.04	0.6	8635	0.061
inter-tribe	whole genome	S.pol+W.lin	143722	0.07	0.6	12757	0.089
inter-tribe	whole genome	S.pol+W.aus	142828	0.07	0.6	11849	0.083
inter-tribe	whole genome	L.min+W.lin	142965	0.07	0.6	13543	0.095
inter-tribe	whole genome	L.min+W.aus	141968	0.07	0.6	12429	0.088
intra-tribe	whole genome	wheat+barley	115940	0.02	1.2	4365	0.038
inter-tribe	whole genome	wheat+B.dis	117055	0.04	1.2	6615	0.057
inter-tribe	whole genome	barley+B.dis	116768	0.04	1.3	6196	0.053
intra-tribe	81 Protein genes	S.pol+L.min	69247	0.03	1.1	420	0.006
intra-tribe	81 Protein genes	W.lin+W.aus	69503	0.03	0.8	633	0.009
inter-tribe	81 Protein genes	S.pol+W.lin	69539	0.04	0.9	819	0.012
inter-tribe	81 Protein genes	S.pol+W.aus	69459	0.04	0.9	682	0.010
inter-tribe	81 Protein genes	L.min+W.lin	69521	0.04	0.9	831	0.012
inter-tribe	81 Protein genes	L.min+W.aus	69468	0.04	0.9	748	0.011
intra-tribe	71 Protein genes	wheat+barley	58607	0.01	1.5	290	0.005
inter-tribe	71 Protein genes	wheat+B.dis	58658	0.03	1.7	1045	0.018
inter-tribe	71 Protein genes	barley+B.dis	58647	0.03	1.7	1034	0.018

a Substitution Rates  =  substitution/alignment length;

b si (Transitional Pairs)  =  AG+CT;

c sv (Transversional Pairs)  =  TA+TG+CA+CG;

d INDELs Ratio  =  INDELs length/alignment length. AG means A is mutated to G and others follow the same rules. S.pol  =  *S. polyrhiza*, L.min  =  *L. minor*, W.lin  =  *W. lingulata*, W.aus  =  *W. australiana,* B.dis  =  *B. distachon*

## Discussion

Next generation sequencing platforms have mainly been used for re-sequencing, SNP analysis, and expression profiling because it has been difficult to develop *de novo* assembly tools for short sequence reads [30]. Whereas re-sequencing or sequencing of related genomes can be very productive for SNP detection and for map-based cloning of mutant alleles, short-read assemblies often fail to detect large INDELs and variable regions in new genomes because technically there is no reference for them. *De novo* assemblies of short reads could cover all insertions, deletions, and rearrangements that would otherwise be incorrectly assembled based on alignments with a reference genome [14]. The pipeline of the SOLiD™ System *de novo* Accessory Tools 2.0, however, has been well adapted to assemble high-coverage SOLiD reads of microbial genomes [31]. Because chloroplasts are even smaller than bacterial genomes, more in the order of large viruses, they represent an exception where such method can be applied. Moreover, we could use paired reads from the same DNA fragment to anchor one end to a contig and the other to a gap that could overlap with other unanchored ends. For this purpose, we used a module Assembly Assistant for SOLiDTM to maximally fill gaps in scaffolds by sufficiently utilizing benefits of these paired ends (http://solidsoftwaretools.com/gf/project/denovo/). Indeed, we got good assemblies by using high quality reads and minimizing non-target DNA from read mixtures. However, interference for contig building arose mainly from long mononucleotide repeats and low complexity sequence. Final mapping of SOLiD reads back to the complete chloroplast genome yielded only 2.5∼12.9% alignment due to 1,000 times smaller genome size than nuclear genome. After comparison of the assembly from computationally selected chloroplast reads with that from total reads, we could show that there is a significant advantage of masking non-chloroplast reads if a related genome sequence is available. Furthermore, without masking, the minimum coverage required to form a contig (coverage cut-off) for Velvet needs to be empirically determined to favor the higher coverage of chloroplast reads over the much lower coverage of nuclei and mitochondria genome sequences to enter the assembly program. Exploration of different computational filters, however, could be used to mask chloroplast sequences instead to favor the assembly of either nuclear or mitochondrial genomic DNA in parallel from the same data set, provided a deep enough genome coverage. Assuming that read length will improve for next generation sequencing platforms as they did for conventional methods in the transition from gel to capillary separation techniques, the major advances in shotgun DNA sequencing are now throughput and computational capacity [32].

It is generally assumed that there is a universal transition bias over transversion, probably as a consequence of the fundamental biochemical basis of mutations [33]. This rule appears to hold quite well in many vertebrate species [34] and it also works very well in the *Pooideae* subfamily as we have calculated here. Surprisingly, this is not the case for the *Lemnoideae* subfamily, where a transition bias is absent. Although there is an exemption of transition bias in coding regions of *Spirodela* and *Lemna*, which could be explained by a selection of nonsynonymous substitutions. If all types of substitutions were to be equal, a 1:2 ratio of transition/transversion would be expected because of two possibilities of transitions (AG+CT) and four of transversions (AT+AC+GT+GC). Excluding nucleotide mutations in coding regions from whole genomes of duckweed chloroplasts, the number of R-values for non-coding region would be very close to 0.5. In such a case, there would be no significant difference between transition and transversion rates. However, in a study of grasshopper pseudogenes a transition/transversion bias was not universal and both substitution rates reached a 1∶1 ratio [35]. Interestingly, transversions could also occur more frequently than transitions in chloroplasts of green algae [36].

Despite the overall high conservation of genome content across different duckweed species, our results demonstrate that substitution rates, insertion and deletion events are more frequent in duckweed chloroplast genomes than in species of the *Pooideae*, especially in non-coding regions (Table 3, Fig. 3). Recent studies also support the observation that *Lemnoideae* have a higher rate of chloroplast sequence evolution relative to *Pistia* and related *Araceae* [37].

Nucleotide substitutions and INDEL mutations are generated during DNA replication or are due to DNA damage [38], [39]. Although the enzymes responsible for the maintenance of chloroplast replication and DNA repair are highly faithful, under certain conditions chloroplasts may have to tolerate some level of oxidative damage that occurs spontaneously due to an abundance of reactive oxygen species from the water-splitting activity of the photosystem [36]. Because duckweeds float on water surface, are fully exposed to sunlight, and produce biomass at such a fast rate, their plastid genomes probably transmit and accumulate mutations more frequently than other plants. Once the genome of *Spirodela* has been sequenced, it will be interesting to analyze its nuclear genes that are involved in DNA replication and repair of the plastid genome and how they have evolved compared to terrestrial slow growing plants.

So far, all phylogeny constructions of *Lemnoideae* have used selected genes or partial regions as markers. However, with sequenced chloroplast genomes of four species in this subfamily and the powerful program to align them, it is possible for the first time to perform whole chloroplast genome phylogenetic analysis. The topology of nodes, all with 100% bootstrap values, conforms to the accepted phylogeny based on extensive analysis from morphology and DNA sequence markers. However, there were two nodes that were problematic with only 42% and 53% bootstrap values in *Wolffia* [9]. Therefore, our results contradict the hypothesis that *Wolffia* arose from a merger of *Wolffiella* and *Lemna*, which was based on the *trnL*-*trnF* marker only [37]. Clearly, the addition of more informative sites from whole genome sequences will improve resolution and confidence in phylogenetic analyses.

In summary, our data gave evidence that next-generation platforms have the capacity to sequence the chloroplast genome at over 1,000 times coverage in just an individual spot on a quadrant slide without plastid purification (Table 2). In order to gain an improved understanding of genome evolution in members of the duckweed subfamily, we generated chloroplast genomes for three species from different genera using *L. minor* as a reference. Our analysis further suggests that (i) gene content is very conserved in duckweeds; (ii) fast nucleotide substitution and abundant INDELs played a key role in the evolution of chloroplast genomes of duckweeds; (iii) duckweed chloroplast genome sequences are very promising to become an elusive single-locus plant barcode for systematic analysis. This information will be critical for the development of a chloroplast transformation system in industrial applications of duckweeds.

## Supporting Information

Table S1 **Amplification primers for genome gap closure and validation.**
(XLS)
